# Supplementary material for: Differences in environmental microbial community responses under rice-crab co-culture and crab monoculture models under cyanobacterial bloom
Source: Front Microbiol. 2024 May 24;15:1327520. doi: 10.3389/fmicb.2024.1327520 (PMC11157002; doi:10.3389/fmicb.2024.1327520)
Supplement: Supplementary file 1 [file Table_1.DOCX]

**Table 1 Network topological parameters of bacterial communities in CMC culture environments**

|  | Nodes | Edges | Average clustering coefficient | Average path length |
| --- | --- | --- | --- | --- |
| RW | 148 | 1831 | 0.642 | 2.006 |
| PW | 148 | 2247 | 0.592 | 0.592 |
| RS | 63 | 218 | 0.586 | 3.328 |
| PS | 60 | 208 | 0.638 | 2.901 |

**Notes:** Nodes represent the number of significant correlations of OTUs that satisfy the following conditions: r > 0.6 or r < −0.6 and statistical significance (FDR-adjusted *P*-values <0.001); Edges represent the numbers of significant correlations between nodes; Average clustering coefficient characterizes the degree of node aggregation; Average path length characterizes the average shortest distance between all node pairs.
