# Supplementary material for: Differences in environmental microbial community responses under rice-crab co-culture and crab monoculture models under cyanobacterial bloom
Source: Front Microbiol. 2024 May 24;15:1327520. doi: 10.3389/fmicb.2024.1327520 (PMC11157002; doi:10.3389/fmicb.2024.1327520)
Supplement: Supplementary file 3 [file Table_3.DOCX]

**Differences in environmental microbial community responses of Chinese mitten crab under rice-crab co-culture and crab monoculture models under cyanobacterial bloom**

Li Tong^1,2+^, Ling Jun^1,2^, Jiang He^1,2^, Yang Min^1,2^, Duan Guoqing^1,2^, Hu Yuting^1,2^, Zhou Huaxing^1,2^, Wang Huan^1,2^, Pan Tingshuang^1,2*^

^1^ Fishery Institute of Anhui Academy of Agricultural Sciences, Hefei 230031, China;

^2^ Key Laboratory of Aquaculture & Stock Enhancement in Anhui Province, Hefei 230031, China

**Abstract**

Cyanobacterial blooms (CBs) present significant challenges to Chinese mitten crab (CMC) culture, posing hazards to the aquatic microbial ecology. However, the current focus on the microbial ecological changes within the CMC culture system under the influence of CBs is somewhat insufficient. There's an urgent need to analyze the microbial ecosystem of the CMC culture system under CBs. This study employed 16S rRNA gene amplicon sequencing to investigate the dynamics of the environmental microbial community in both the rice-crab co-culture (RC) and crab monoculture (CM) models. The results revealed that cyanobacteria reached high levels in the CM water in July, while they began to increase in the RC water in August. Notably, OTU147 (uncultured bacterium g_*Planktothrix NIVA-CYA 15*), identified as the dominant taxon associated with CBs, showed a significant linear relationship with TP, NO_2_^-^-N, and the N:P ratio. TP, TN, NO_2_^-^-N, and COD_Mn_ had a more pronounced impact on the structure of bacterial communities and cyanobacterial taxa in the water. The bacterial community structure involved in carbon metabolism displayed temporal succession in the water. The co-occurrence network of the bacterial community primarily consisted of *Chloroflexi*, *Proteobacteria*, and *Firnicutes* in the sediment, and *Actinobacteria*, *Proteobacteria*, *Chloroflexi*, and *Bacteroidota* in the water. In contrast, the co-occurrence network included different peripheral species in the sediment and water. Keystone species were predominantly represented by OTU22 (*uncultured actinobacterium g_ hgcI clade*) and OTU12 (uncultured *Opitutae bacterium* g_ norank) in the RC water, and by OTU25 (*uncultured bacterium g_ Limnohabitans*) in the CM water. TP, TN, NO_2_^-^-N, and COD_Mn_ were identified as the primary environmental factors influencing these keystone taxa within the culture water. In conclusion, this study on the microbial ecology of the CMC culture system under the influence of CBs provides valuable insights that can be instrumental in subsequent management efforts.

**Keywords: cyanobacterial blooms (CB), Chinese mitten crab, crab culture models, bacterial community, environmental factors, co-occurrence network, keystone taxa, carbon metabolism**

**Introduction**

The Chinese mitten crab (*Eriocheir sinensis*, CMC) is a vital economic species in China, with an annual output that has ranged between 700,000 and 800,000 tons in recent years. However, challenges including extreme weather, eutrophication of culture water, and the decay of aquatic plants have led to frequent occurrences of cyanobacterial blooms (CBs) within the CMC culture system. The state of the culture environment is critical for the successful progress of CMC culture, and CBs pose a significant threat to the industry's development. Microbial communities, which can rapidly respond to environmental changes, play a pivotal role in predicting and characterizing aquaculture diseases and the quality of culture systems (Huang et al., 2018; Xiong et al., 2015; Xiong et al., 2014). For instance, deteriorating water quality can result in the overgrowth and reproduction of *Vibrio* in the water, leading to disease outbreaks and even mass shrimp fatalities (Xiong et al., 2014; Zhou et al., 2012). The occurrence of CBs can trigger variations in the bacterial community structure of the water, highlighting the microbial community's role as a bridge connecting the environment and aquatic animals (Huang et al., 2018). Investigating changes in the microbial community in the CMC culture system will enhance our ability to conduct aquaculture activities successfully.

CBs can induce dynamic changes in both benthic and planktonic microbial communities in the aquatic environment. Understanding how microbial communities respond to the emergence and dissipation of CBs is crucial for preventing and managing these blooms and mitigating the associated risks. Currently, there is limited research on how microorganisms involved in environmental material cycling and energy flow within CMC culture ponds respond to CBs. Most research has focused on characterizing changes in the composition and structure of the microbial community within the CMC culture environment during CB events, such as significant alterations in the water's bacterial community structure at different stages of CBs (Zhu et al., 2019). Several environmental factors significantly influence cyanobacterial dynamics in CMC culture environments. Excessive nutrient levels, particularly nitrogen and phosphorus, promote cyanobacterial growth, and higher temperatures accelerate their proliferation. These environmental factors do not act in isolation; they interact and collectively affect cyanobacterial bloom formation in crab culture environments. For instance, research by Li et al. (2019) demonstrated that the N:P ratio, water temperature (WT), and total phosphorus (TP) were key environmental drivers influencing the complex development of cyanobacterial communities in crab ponds in Lake Guchenghu, China. It is essential to further analyze how the bacterial community in the CMC culture system responds to changes induced by CBs. Such analysis will facilitate the development of strategies to prevent and mitigate the harmful effects of CBs.

Microbial communities in the natural environment do not exist independently; instead, they form complex networks through predation, competition, and mutualistic symbiosis to maintain the stability of their structure and function (Faust and Raes, 2012). The importance of maintaining microbial community networks in aquatic environments is self-evident. Co-occurrence network analysis has been used to explain microbial community interactions, and related studies have found that keystone species contribute to regulating the microbial community structure and function (Ma et al., 2016). For example, when compared to irrigation canals, sediment microbial communities in aquatic ponds exhibit more stable interactions, with microorganisms of relatively low relative abundances potentially acting as key players in sediment ecosystems (Xu et al., 2022). Microbial communities in different types of CMC culture systems respond differently to cyanobacteria, and various CBs determine which microorganisms survive, establish specific microbial metabolic pathways, and influence their activities (Li et al., 2019; Ma et al., 2021). Therefore, our objective is to gain insights into the associations between microbial communities in different types of systems and their network co-occurrence patterns under CB conditions, aiming to elucidate the co-occurrence patterns of microbial communities and identify keystone species within these ecosystems.

In this study, the impact of CB on bacterial communities in both the water and sediment of CMC culture systems, specifically the rice-crab co-culture and crab monoculture models, was assessed using 16S rRNA gene amplification and sequencing. The primary objectives were to: 1) Investigate the alterations in the composition of microbial communities in both the water and sediment of culture environments under the influence of CB. 2) Characterize the differentiation of bacterial communities resulting from physicochemical factors between different CMC culture modes under CB. 3) Examine the variations in carbon metabolism within microbial communities in both the water and sediment under CB. 4) Compare the differences in microbial co-occurrence patterns between the two types of culture environments under the influence of CB.

**Materials and methods**

**Experimental materials**

This experiment was conducted in the Tengfei Aquatic Products Professional Cooperative Juvenile Crab Cultivation Core Demonstration Area, located in the Xianzhou district of Xuancheng, Anhui province, China (latitude: 118°45' N, longitude: 31°11' S). The experiment was divided into two models: Rice-crab co-culture (RC) and crab monoculture models (CM).

Basic Information: In the experimental ponds, there had been CBs occurrence in the past years, and it is well known that the probability of CB occurrence is very high in these ponds. Under this background, we carried out RC in three of these ponds and CM in the adjacent three ponds. CBs in the CM system occurred in July, while in the RC system occurred in August. The whole sampling process can also be divided into non-BC outbreak phase, BC outbreak phase and BC recession phase. Each pond was covered an area of approximately 1,334 m². On March 5, 2022, Alternanthera philoxeroides was planted throughout the entire pond in the CM model, whereas in the RC model, it was only planted in the peripheral trench. On May 7, 2022, rice (Brand: Youliangyou 2152) was transplanted using rice planting technology in the RC group. Megalopa larvae were released after the rice transplant in both modes, with each field stocked with 2.4×10^5^ megalopa, each averaging about 0.008 g in weight. The rice was harvested at the end of October.

**Sample collection**

Sediment (S) and water (W) samples were collected from June to mid-November, excluding October, for subsequent monitoring. Sampling locations for water or sediment samples of each timepoint were repetition in each pond. Physicochemical factors of the culture water were continuously monitored. One liter of water from the middle layer of the pond was collected using a diagonal two-point method. Subsequently, microorganisms in the water were captured after passing through a 2 μm filter membrane. These microorganisms were then placed in sterilized centrifuge tubes, stored in refrigerators at -80°C for subsequent DNA extraction, and finally used for 16S rRNA gene sequencing analysis. Sediment samples, with a surface layer height of 5 cm, were collected using the diagonal two-point method. These samples were placed in sterilized centrifuge tubes and stored in a refrigerator at -80°C for subsequent DNA extraction for 16S rRNA gene sequencing analysis and heavy metal index detection.

The water quality index detection methods are as follows: Ammonia nitrogen (NH4^+^-N) was detected using Nessler reagent spectrophotometry. Nitrite nitrogen (NO_2_^-^-N) was detected using spectrophotometry. Nitrate nitrogen (NO_3_^-^-N) was detected using ultraviolet spectrophotometry. Soluble orthophosphate (TP) was detected using molybdenum antimony spectrophotometry. Total nitrogen (TN) was determined using ultraviolet spectrophotometry after digestion with alkaline potassium persulfate. Total phosphorus was detected using ammonium molybdate spectrophotometry. Chemical oxygen demand (COD_Mn_) was determined using permanganate as an oxidant.

The methods for detecting heavy metals, pH, and organic matter in sediment are as follows: Arsenic (As) content was tested in accordance with the DZ/T0279.13-2016 standard. Mercury (Hg) content was tested following the DZ/T0279.17-2016 standard. Organic matter content (TOC) was determined based on the DZ/T0279.27-2016 standard. pH was tested as per the DZ/T0279.34-2016 standard. Cadmium (Cd) content was tested following the DZ/T0279.5-2016 standard. Chromium (Cr), Copper (Cu), Nickel (Ni), Lead (Pb), and Zinc (Zn) contents were analyzed in accordance with the HJ780-2015 standard.

**DNA extraction and high-throughput sequencing**

The SDS method was employed to extract total DNA from water and sediment samples. DNA quality and concentration were assessed using a Nanodrop ND-2000 (Nano Drop Technologies, USA). For amplification of the V3-V4 region of the 16S rRNA gene, primer sets 341F (5'-CCTAYGGGRBGCASCAG-3') and 806R (5'-GGACTNNGGGTATCTAAT-3') were selected. The amplification was performed using a Bio-Rad T 100 thermocycler (Bio-Rad, USA). The PCR product of each sample was purified using a PCR fragment purification kit (Thermo Scientific, USA), and the concentration of the purified PCR product was measured with an Agilent 2100 bioanalyzer (Agilent, USA). A library was created by pooling equimolar amounts of PCR amplicons from each sample using the TruSeq DNA PCR-Free Library Preparation Kit (Illumina, USA). The library was quantified using a Qubit 2.0 fluorometer (Life Technologies, USA) and sequenced using the NovaSeq 6000 system (Illumina, USA). The sequencing data have been deposited as accession number PRJNA1030144 in the NCBI BioProject database.

**The microbial community analysis**

The Similarity Percentages (SIMPER) method was utilized to analyze the primary differences in bacterial community composition by using PAST4 software. To investigate the impact of physicochemical factors on the bacterial community structure in both sediment and water, we employed the vegan package within the R environment for Redundancy Analysis (RDA). Furthermore, we used the varpart function (VPA) from the "vegan" package in the R environment to assess the importance of environmental variables throughout the entire culture process.

Phylogenetic investigation of communities by reconstruction of unobserved states (PICRUSt2) (https://github.com/picrust/picrust2) was used to predict the carbon metabolism function of bacterial community in the water, and further analyzed by comparing with KEGG orthology. To assess structural changes in gene functions related to carbon metabolism, we utilized Non-Metric Multidimensional Scaling (NMDS). Additionally, we used Analysis of Similarities (ANOSIM) to evaluate structural differences in gene functions related to carbon metabolism at adjacent CB stages.

To explore the differences in co-occurrence patterns of bacterial communities under different culture modes, spearman correlation was employed to examine the relationships between OTUs in sediment and water, respectively. We applied Spearman correlations to filter nodes and edges with significant associations using the “Hmisc” package (Harrell et al., 2019) within the R environment. To calculate network topology parameters, including nodes, edges, clustering coefficient, average path length, degree, closeness centrality, betweenness centrality, and eigencentrality, we used Gephiv 0.9.2. Furthermore, to analyze the differences in node topology parameters within bacterial community networks of the two different culture modes, we employed the Wilcox.test in the “ggsigif” package (Ahlmann-Eltze and Patil, 2021) in the R environment.

To further explore the keystone species within the microbial community in both sediment and water, we calculated the within-module connectivity (Zi) and inter-module connectivity (Pi) of the bacterial community in sediment and water using the "microeco" package in the R environment (Liu et al., 2021). We performed Spearman analysis using the "corrplot" package (Wei et al., 2017) to assess the physicochemical factors that impact these keystone species.

**Results**

**Bacterial community succession characteristics under different CMC culture modes**

At the phylum level, the bacterial communities in the sediment under both culture modes were primarily composed of Chloroflexi (CM: 27.13% vs. RC: 23.52%), *Proteobacteria* (13.21% vs. 14.86%), and *Firmicutes* (CM: 11.53% vs. RC: 12.56%) **(Figure 1A)**. In contrast, the bacterial communities in the water were primarily composed of *Acidobacteriota* (CM: 27.90% vs. RC: 33.06%), *Proteobacteria* (CM: 21.86% vs. RC: 27.74%), and *Cyanobacteria* (CM: 29.30% vs. RC: 13.04%) **(Figure 1B)**. Notably, the abundance of *Cyanobacteria* reached a peak of 52.91% in the CM system in July, while it began to increase in the RC system in August, with a relative abundance of 47.87% **(Figure 1B)**. At the genus level, the bacterial communities in the sediment were predominantly composed of *norank Anaerolineaceae* (CM: 10.42% vs. RC: 8.67%), *Bacillus* (CM: 3.04% vs. RC: 3.41%), and *norank Vicinamibacterales* (CM: 2.89% vs. RC: 3.08%) **(Supplementary Figure 1)**. Their composition remained relatively stable over time, and their relative abundances were similar between the two culture modes **(Supplementary Figure 1)**. In contrast, the bacterial communities in the water were mainly composed of *Planktothrix NIVA.CYA 15* (CM: 22.56% vs. RC: 10.35%), *hgcI clade* (CM: 6.57% vs. RC: 6.15%), *Mycobacterium* (CM: 4.26% vs. RC: 7.03%), and *Rhodoluna* (CM: 1.81% vs. RC: 5.93%) **(Supplementary Figure 1)**.

According to the SIMPER analysis, twelve OTUs, including OTU685 (*Bacillus selenatarsenatis*), OTU374 (unclassified f_*Nocardioidaceae*), and OTU2690 (uncultured gamma proteobacterium g_norank f_*Steroidobacteriaceae*), were identified as the primary bacterial communities responsible for the differences between the two sediment modes **(Figure 2A)**. Additionally, eight OTUs, such as OTU147 (uncultured bacterium g_*Planktothrix NIVA-CYA 15*), OTU5 (uncultured bacterium g_*Rhodoluna*), and OTU1 (uncultured Canditatus *Planktophila sp.*), were the main bacterial taxa contributing to the distinctions between the two water modes **(Figure 2B)**.

**Physicochemical factor driven variation of bacterial communities under different CMC culture modes**

TOC, Cd, Cr, Cu, Ni, and Zn exhibited a significant impact on the structure of sediment bacterial communities. In contrast, TP, TN, NO_2_^-^-N, and COD_Mn_ had a more pronounced influence on the structure of bacterial communities in the water (Figure 3). Further analysis revealed that TP, NO_2_^-^-N, and COD_Mn_ also had a significant impact on cyanobacterial taxa **(Figure 3)**. When assessing the variation in sediment bacterial communities, it was found that heavy metals and TOC together explained 8.94% of the variance, with heavy metals accounting for 7.4% and TOC contributing 1.54% in the VPA analysis **(Figure 3)**. On the other hand, the explanatory power of TP on the variation of water bacterial communities was notably high, at 21.61%, surpassing the impact of other physicochemical factors and COD_Mn_ **(Figure 3A)**. The cumulative explanatory effect of these physicochemical factors on cyanobacterial taxa in the water amounted to 29.09%, with N-related physicochemical factors explaining 7.24% and COD_Mn_ contributing 1.10% **(Supplementary Figure 2)**. Furthermore, a linear relationship was observed between OTU147 (uncultured bacterium g_*Planktothrix NIVA-CYA 15*) and TP, NO_2_^-^-N, and the N:P ratio **(Supplementary Figure 2)**.

**Differences of carbon metabolic of bacterial communities under the two culture modes**

Using PICRUSt2, a total of 284 genes related to carbon metabolic functions were identified within the bacterial communities of sediments throughout the entire process. Additionally, there were 278 genes associated with carbon metabolic functions within the bacterial communities of water. In total, 365 KEGG orthology genes related to carbon metabolism were identified **(Supplementary Table 1)**. Genes related to carbon metabolism, with relative abundances exceeding 30,000, were selected for further analysis. Through NMDS analysis, the structure of carbon metabolism-related genes in sediment bacterial communities exhibited a scattered distribution **(Figure 4A)**. Subsequent analysis using PERMANOVA indicated that there was no significant difference in the bacterial communities of carbon metabolism-related genes between the two culture modes over time (R^2^ = 0.06072, *P* = 0.1648). On the other hand, NMDS analysis revealed that the structure of carbon metabolism-related genes in water bacterial communities displayed characteristics of succession over time **(Figure 4B)**. Further analysis using PERMANOVA showed a significant difference in the bacterial communities of carbon metabolism-related genes between the two culture modes over time (R^2^ = 0.10592, *P* < 0.001).

**Network characteristics of bacterial communities under different CMC culture modes**

The sediment bacterial community network under the RC mode comprised 63 nodes and 218 edges, while the CM mode had relatively fewer nodes and edges **(Figure 5A)**. Additionally, the average clustering coefficient of the RC mode was higher than that of the CM mode, while the average path length was lower than that of the CM mode **(Table 1)**. The positive correlation ratio of sediment bacterial communities in the RC mode was 78.44%, which was higher than that of the CM mode **(Figure 5A)**. In both culture modes, the predominant components were *Chloroflexi*, *Proteobacteria*, *Firnicutes*, and *Actinobacteriota* **(Figure 5A)**. Significant differences in closeness centrality between nodes in the bacterial community networks were observed between the two culture modes **(Supplementary Figure 3)**. ZiPi analysis indicated that microbial communities in both culture modes were primarily composed of peripherals **(Figure 5C)**.

The water bacterial community network under the RC mode consisted of 148 nodes and 2831 edges, while the CM mode had relatively fewer edges **(Figure 5B)**. Additionally, the average clustering coefficient of the RC mode was lower than that of the CM mode, while the average path length was higher than that of the CM mode **(Table 1)**. The positive correlation ratio of water bacterial communities in the RC mode was 59.59%, which was lower than that of the CM mode. In both modes, the primary components were *Actinobacteria*, *Proteobacteria*, *Chloroflexi*, and *Bacteroidota* **(Figure 5B)**. Significant differences in degree and closeness centrality between nodes in the bacterial community networks were observed between the two modes **(Supplementary Figure 3)**.

ZiPi analysis indicated that microbial communities in both modes were primarily composed of peripherals **(Figure 5C)**. In the RC mode, microbial taxa with network hubs and connects were considered keystone taxa, including OTU22 *(uncultured actinobacterium g_ hgcI clade*) and OTU12 (*uncultured Opitutae bacterium g_ norank*) **(Figure 6)**. In the CM mode, microbial taxa with connects were also keystone taxa, mainly including OTU25 (*uncultured bacterium g_ Limnohabitans*) **(Figure 6)**. Furthermore, a correlation analysis between keystone taxa and environmental factors revealed that TP, TN, NO_2_^-^-N, and COD_Mn_ in pond water were factors affecting keystone taxa. These same physicochemical factors also influenced keystone taxa in the RC mode water **(Figure 6)**.

**Discussion**

The trend of *Planktothrix* blooms in CMC culture systems has been increasingly severe. Pond culture systems, with *Planktothrix* dominating, experienced a bloom as early as July. Typically, *Planktothrix* blooms are known to occur in late summer (Ma et al., 2021; Millie et al., 2009). This trend highlights the advancing nature of CB and their potentially negative impacts on CMC culture. To our surprise, the RC system demonstrated lower bloom intensity and timing compared to the CM mode **(Supplementary Figure 1)**. This suggests that the RC system possesses more robust self-regulation, and its ecological value is enhanced by employing rice as an alternative to *Alternanthera philoxeroides* in CMC culture.

CB has a significant influence on the composition and variation of bacterial communities in CMC culture water **(Figure 1)**. This observation aligns with findings in CMC culture systems in lakes (Li et al., 2019). Notably, the bacterial community composition in CMC culture water exhibits significant variations under CB conditions, and different functional taxa display distinct succession patterns. For instance, in our study, *Planktothrix*, a dominant *cyanobacterium* at the genus level, proliferated in the CMC culture system. OTU147, belonging to *Planktothrix*, emerged as the dominant taxon responsible for the differences in water composition between the two culture modes. It has been reported that toxic *Planktothrix* can lead to surface water odor pollution (Su et al., 2017). Thus, the impact of OTU147 (uncultured bacterium g_*Planktothrix NIVA-CYA 15*) on CMC farming is significant and should not be underestimated **(Figure 2)**.

One of the concerning aspects of CBs is their potential to cause the accumulation of pathogenic bacteria. The occurrence of *Planktothrix* has led to the accumulation of *Mycobacterium* **(Figure 2)**, posing a health threat to humans (Dong et al., 2021) through water exposure. Therefore, considering the advancing *Planktothrix* blooms and the concurrent increase in pathogenic bacteria, it is imperative to emphasize the secondary hazards associated with *Planktothrix* blooms in CMC culture. Preventative and control measures are of paramount importance.

Several environmental factors, such as nutrients, have been shown to influence *Planktothrix* bloom (Cheng et al., 2022). In our study, we found that TP, NO_2_^-^-N, and COD_Mn_ had a significant impact on the bacterial community composition in the water **(Figure 3)**. Furthermore, we observed a significant positive correlation between the *Planktothrix* bloom and the levels of TN, TP, and COD_Mn_ **(Supplementary Figure 2)**. This finding aligns with a study conducted in crab ponds in Guchenghu, where the N:P ratio, WT, and TP were identified as significant factors affecting cyanobacterial development (Li et al., 2019), which is in line with our research.

The substantial input of nitrogen and phosphorus into the water has been identified as a key factor in promoting *Planktothrix* formation (Davis et al., 2015; Wang et al., 2023). In our study, we noticed that the TP content in RC water was lower than that in CM water during the same time period, while the intensity of *Planktothrix* blooms in the RC system was lower than that in the CM culture system **(Supplementary Figure 1)**. This suggests that, in RC models, rice roots may have a greater tendency to utilize available phosphorus compared to *Alternanthera philoxeroides*.

*Planktothrix*, as carbon-fixing microorganisms, plays a critical role in converting atmospheric carbon into organic carbon in the water (Zhang et al., 2020). Our further analysis indicated that COM drives the succession of *Planktothrix* blooms. Prior to the rice harvest, the COD_Mn_ content in the RC system was lower than that in the CM system. During this time, CB in the CM system was more severe than in the RC system. However, after the rice harvest, the COD_Mn_ content in the RC system increased compared to the CM system. This change could be attributed to the reduced nutrient absorption by rice roots in the water of the RC system and the presence of remaining roots in the culture system, leading to increased eutrophication and organic matter in the system. However, as temperatures decreased during this period, the intensity of CB in the culture system tended to decline, and the microbial community that relies on organic matter was no longer dominated by *Planktothrix*. As a result, the adverse impact on the culture system was relatively reduced. Thus, we conclude that *Planktothrix* blooms in the CMC culture system are linked to increases in physicochemical factors such as TP and COD_Mn_ **(Supplementary Figure 2)**.

The RC system exhibits more intricate relationships between bacterial communities in sediment and water compared to the CM mode. This complexity may arise from the more intricate ecological environment created by rice cultivation in the RC system. Higher clustering coefficients and shorter average path lengths are indicative of more pronounced small-world effects, as outlined by Collins and Chow (1998). Our research findings suggest that the bacterial community in sediment, forming a small-world network, is more stable under the RC mode. In contrast, the bacterial community in water, also forming a small-world network, appears to be relatively fragile. The positive correlation ratio between sediment bacterial communities under the RC mode is significantly higher than that under the CM mode **(Figure 5)**. This suggests that cooperation within sediment bacterial communities under the RC mode is more robust, and frequent collaboration among bacteria may enhance their ability to adapt to environmental changes. Conversely, the negative correlation ratio between water bacterial communities under the RC mode is higher than that under the CM mode **(Figure 5)**. This indicates that competition is more intense within water bacterial communities under the RC mode. In summary, our data reveals that, when compared to the CM modes, the complex ecosystems of the RC mode can lead to more stable microbial networks, providing a degree of resilience against *Planktothrix* blooms.

In addition, our data shows that *Planktothrix* blooms have an impact on the bacterial community structure and carbon metabolism **(Figure 4)**. Several keystone taxa, primarily consisting of OTU22 (*uncultured actinobacterium g_ hgcI clade*) and OTU12 (*uncultured Opitutae bacterium g_ norank*) in our study **(Figure 6)**, require carbon and nitrogen sources for their growth. Members of *Opitutae* contain nitrogen fixation genes (Wertz et al., 2012), while *Roseiflexus* requires carbon sources for growth and is strictly phototrophic (Wertz et al., 2012). *Fluviicola* may indicate good water quality (Ji et al., 2018) and exhibit efficient performance in nitrogen and carbon removal (Chen et al., 2020). In RC mode, the microbial taxa serving as keystone species are predominantly composed of OTU25 (*uncultured bacterium g_ Limnohabitans*) **(Figure 6)**, among which *Limnohabitans* plays a role in nitrogen cycling and cyanobacteria degradation (Zhu et al., 2020). The taxon of norank_f_JG30-KF-CM45 is involved in denitrification.TN, NO_2_^-^-N, and COD_Mn_ are factors affecting the keystone taxa in CMC culture water **(Figure 6)**. This indicates that the keystone microbial taxa in CMC culture water actively participate in carbon and nitrogen cycling processes. These keystone taxa can compete for additional carbon and nitrogen sources in the water to support their growth, ensuring the stable operation of the entire aquaculture system. Moreover, these keystone taxa possess the ability to degrade *cyanobacteria*, thereby mitigating the harm caused by cyanobacterial blooms to some extent.

**Conclusion**

In summary, our research offers valuable insights into the mechanisms driving microbial community changes in the CMC culture system during *Planktothrix* blooms from a microbial ecology perspective. We observed that both the timing and intensity of *Planktothrix* blooms in the RC mode were lower than those in the CM mode. The RC system exhibits more complex relationships among bacterial communities when compared to the CM mode. The occurrence of *Planktothrix* blooms in the CMC culture system is associated with increased physicochemical factors, such as TP and COD_Mn_, and alterations in the bacterial community structure related to carbon metabolism. Furthermore, the keystone taxa identified in our study are reliant on carbon and nitrogen sources for their growth and possess the capacity to degrade cyanobacteria. This research offers valuable environmental microbial data to support the management of cyanobacterial outbreaks in CMC culture.

**AUTHOR CONTRIBUTIONS**

LT, LJ, YM, JH, P-TS conceptualized, collected, analyzed data and wrote the original draft. LT, P-TS, D-GQ, H-YT, Z-HX and WH edited the manuscript draft. LT and JH conceptualized the review. All authors contributed to the article and approved the submitted version.

**FUNDING**

This work was Supported by the earmarked fund for CARS-48.

**ACKNOWLEDGMENTS**

Thanks to all participants of Xuanzhou District Fisheries Technology Extension Center, Xuancheng City, Anhui Province, China in conducting the experiment.

**Reference**

Ahlmann-Eltze, C., and Patil, I. (2021). ggsignif: R Package for Displaying Significance Brackets for'ggplot2'. doi:[10.31234/osf.io/7awm6](http://dx.doi.org/10.31234/osf.io/7awm6)

Chen, S., Chen, W., Wu, S., Wang, X., and Wang, J. (2020). Effects of external resistance on denitrification and carbon removal performance of a microbial fuel cell used in simulated groundwater. *Fresenius Environmental Bulletin, 29*(7), 5464-5471.

Cheng, J., Ma, L., Zhao W., Liu Y. (2022). Effects of Environmental Factors on the Growth and 2-MIB Production of *Planktothrix* sp.. *Journal of Hydroecology, 43*(2), 7. doi: 10.15928/j.1674-3075.202008180236

Collins, J., Chow, C. (1998). It's a small world. Nature 393, 409–410 (1998). Doi: 10.1038/30835

Dong, W., Nie, X., Zhu, H., Liu, Q., Shi, K., You, L., et al. (2021). Mycobacterial fatty acid catabolism is repressed by FdmR to sustain lipogenesis and virulence. *Proceedings of the National Academy of Sciences, 118*(16), e2019305118. doi:10.1073/pnas.2019305118

Faust, K., and Raes, J. (2012). Microbial interactions: from networks to models. *Nat Rev Microbiol, 10*(8), 538-550. doi:10.1038/nrmicro2832

Harrell Jr, F. E., and Harrell Jr, M. F. E. (2019). Package ‘hmisc’. *CRAN2018, 2019*, 235-236. https://cran.uib.no/web/packages/Hmisc/Hmisc.pdf

Huang, F., Pan, L., Song, M., Tian, C., and Gao, S. (2018). Microbiota assemblages of water, sediment, and intestine and their associations with environmental factors and shrimp physiological health. *Applied Microbiology and Biotechnology, 102*(19), 8585-8598. doi:10.1007/s00253-018-9229-5

Ji, B., Qin, H., Guo, S., Chen, W., Zhang, X., and Liang, J. (2018). Bacterial communities of four adjacent fresh lakes at different trophic status. *Ecotoxicology and Environmental Safety, 157*(8), 388-394. [doi: 10.1016/j.ecoenv.2018.03.086](https://doi.org/10.1016/j.ecoenv.2018.03.086)

Li, H., Chen, H., Gu, X., Mao, Z., Zeng, Q., and Ding, H. (2019). Dynamics of cyanobacteria and related environmental drivers in freshwater bodies affected by mitten crab culturing: a study of lake guchenghu, china. *Water, 11*(12), 2468. doi: 10.3390/w11122468

Liu, C., Cui, Y., Li, X., and Yao, M. (2021). microeco: an R package for data mining in microbial community ecology. *FEMS Microbiology Ecology, 97*(2), fiaa255. doi: 10.1093/femsec/fiaa255

Ma, B., Wang, H., Dsouza, M., Lou, J., He, Y., Dai, Z., et al. (2016). Geographic patterns of co-occurrence network topological features for soil microbiota at continental scale in eastern China. *ISME J, 10*(8), 1891-1901. doi:10.1038/ismej.2015.261

Ma, Q., Wang, Y., Li, C., Fan P., Zhou, J., Fang, W. (2021). Influence of cyanobacterial bloom dominated by *Planktothrix* sp. and *Cylindrospermopsis raciborskii* on microflora structure of intestine, gill and culture enviroment of cultured *Eriocheir sinensis*. *Mairne Fisheries. 43*(5), 595-606. doi:10.3969/j.issn.1004-2490.2021.05.009

Millie, D. F., Fahnenstiel, G. L., Dyble Bressie, J., Pigg, R. J., Rediske, R. R., Klarer, D. M., et al. (2009). Late-summer phytoplankton in western Lake Erie (Laurentian Great Lakes): bloom distributions, toxicity, and environmental influences. *Aquatic Ecology, 43*(4), 915-934. doi:10.1007/s10452-009-9238-7

Su, M., Jia, D., Yu, J., Vogt, R. D., Wang, J., An, W., and Yang, M. (2017). Reducing production of taste and odor by deep-living cyanobacteria in drinking water reservoirs by regulation of water level. *Science of The Total Environment, 574*, 1477-1483. doi:https://doi.org/10.1016/j.scitotenv.2016.08.134

Wei, T., Simko, V., Levy, M., Xie, Y., Jin, Y., and Zemla, J. (2017). Package ‘corrplot’. *Statistician, 56*(316), e24. https://github.com/taiyun/corrplot

Wertz, J. T., Kim, E., Breznak, J. A., Schmidt, T. M., and Rodrigues, J. L. M. (2012). Genomic and physiological characterization of the *verrucomicrobia* isolate *diplosphaera colitermitum* gen. nov., sp. nov., reveals microaerophily and nitrogen fixation genes. *Applied and Environmental Microbiology, 78*(5), 1544-1555.

Xiong, J., Wang, K., Wu, J., Qiuqian, L., Yang, K., Qian, Y., and Zhang, D. (2015). Changes in intestinal bacterial communities are closely associated with shrimp disease severity. *Applied Microbiology and Biotechnology, 99*(16), 6911-6919. doi:10.1007/s00253-015-6632-z

Xiong, J., Zhu, J., and Zhang, D. (2014). The application of bacterial indicator phylotypes to predict shrimp health status. *Applied Microbiology and Biotechnology, 98*(19), 8291-8299. doi:10.1007/s00253-014-5941-y

Xu, M., Xu, R., Shen, X., Gao, P., Xue, Z., Huang, D., et al. (2022). The response of sediment microbial communities to temporal and site-specific variations of pollution in interconnected aquaculture pond and ditch systems. *Science of The Total Environment, 806*, 150498. doi: 10.1016/j.scitotenv.2021.150498

Zhang, X., Ye, X., Chen, L., Zhao, H., Shi, Q., Xiao, Y., et al. (2020). Functional role of bloom-forming cyanobacterium *Planktothrix* in ecologically shaping aquatic environments. *Science of The Total Environment, 710*, 136314. doi: 10.1016/j.scitotenv.2019.136314

Zhou, J., Fang, W., Yang, X., Zhou, S., Hu, L., Li, X., et al. (2012). A nonluminescent and highly virulent Vibrio harveyi strain is associated with “bacterial white tail disease” of Litopenaeus vannamei shrimp. *PLoS One*, 7(2), e29961. doi: 10.1371/journal.pone.0029961

Zhu, C., Zhang, J., Nawaz, M. Z., Mahboob, S., Al-Ghanim, K. A., Khan, I. A., et al. (2019). Seasonal succession and spatial distribution of bacterial community structure in a eutrophic freshwater Lake, Lake Taihu. *Science of The Total Environment, 669*, 29-40. doi: 10.1016/j.scitotenv.2019.03.087

Zhu. J.,Qin. H., Sun. Q., Wang. B., Gao. R., Guo. R., Li. W. (2020). Microbial diversity and influencing factors in a small watershed in winter.Chinese. *Journal of Environmental Science*. 41(11):5016-5026

**Legends**

**Figure 1** The composition of bacterial communities in the CMC culture environment at phylum level. (A) Sediment; (B) Water

**Figure 2** Similarity of bacteria community between RC and CM culture modes based on SIMPER analysis. (A) Sediment; (B) Water

**Figure 3** Impact of environmental factors on the bacterial community structure of the CMC culture environment based on RDA and VPA analysis. A: Sediment; B: Water

**Figure 4** The structure of bacteria community function of Carbon metabolism.

**Figure 5** The co-occurrence network of bacterial community in the CMC culture environment. A: network graphs of sediment; B: network graphs of water; C: ZiPi plot. **Notes:** In Zipi plots, nodes were categorized into four functional types based on their topological characteristics. These categories included: Connectors: Nodes with high connectivity within two modules (Zi>2.5 and Pi<0.6). Module hubs: Nodes with high connectivity between two modules (Zi<2.5 and Pi>0.62). Network hubs: Nodes with high connectivity (Zi>2.5 and Pi>0.62). Peripherals: Nodes that do not exhibit high connectivity (Zi<2.5 and Pi<0.62) either within or between modules.

**Figure 6** The relationships of keystone species of bacterial community and environmental factors in the CMC culture water. Notes: *denotes a correlation with significance *P* < 0.05, ** denotes a correlation with significance *P* < 0.01, ** denotes a correlation with significance *P* < 0.001.

**Supplementary Figure 1** Pheatmap of bacterial communities in the CMC culture environment at genus level. (A) Sediment; (B) Water

**Supplementary Figure 2** The relationships of cyanobacterial taxa and environmental factors in the CMC culture water. (A) RDA analysis; (B) VPA analysis; (C) the change of relative abundance of OTU147; (D) correlation analysis of OTU147 and environmental factors

**Supplementary Figure 3** Node-level topological features of the bacterial network in the CMC culture environment. (A) Sediment; (B) Water

**Supplementary Table 1** Genes related to carbon metabolic functions identified within the bacterial communities in the CMC culture environment by using PICRUSt2
