# Supplementary figures and images for: Differences in environmental microbial community responses under rice-crab co-culture and crab monoculture models under cyanobacterial bloom
Source: Front Microbiol. 2024 May 24;15:1327520. doi: 10.3389/fmicb.2024.1327520 (PMC11157002; doi:10.3389/fmicb.2024.1327520)

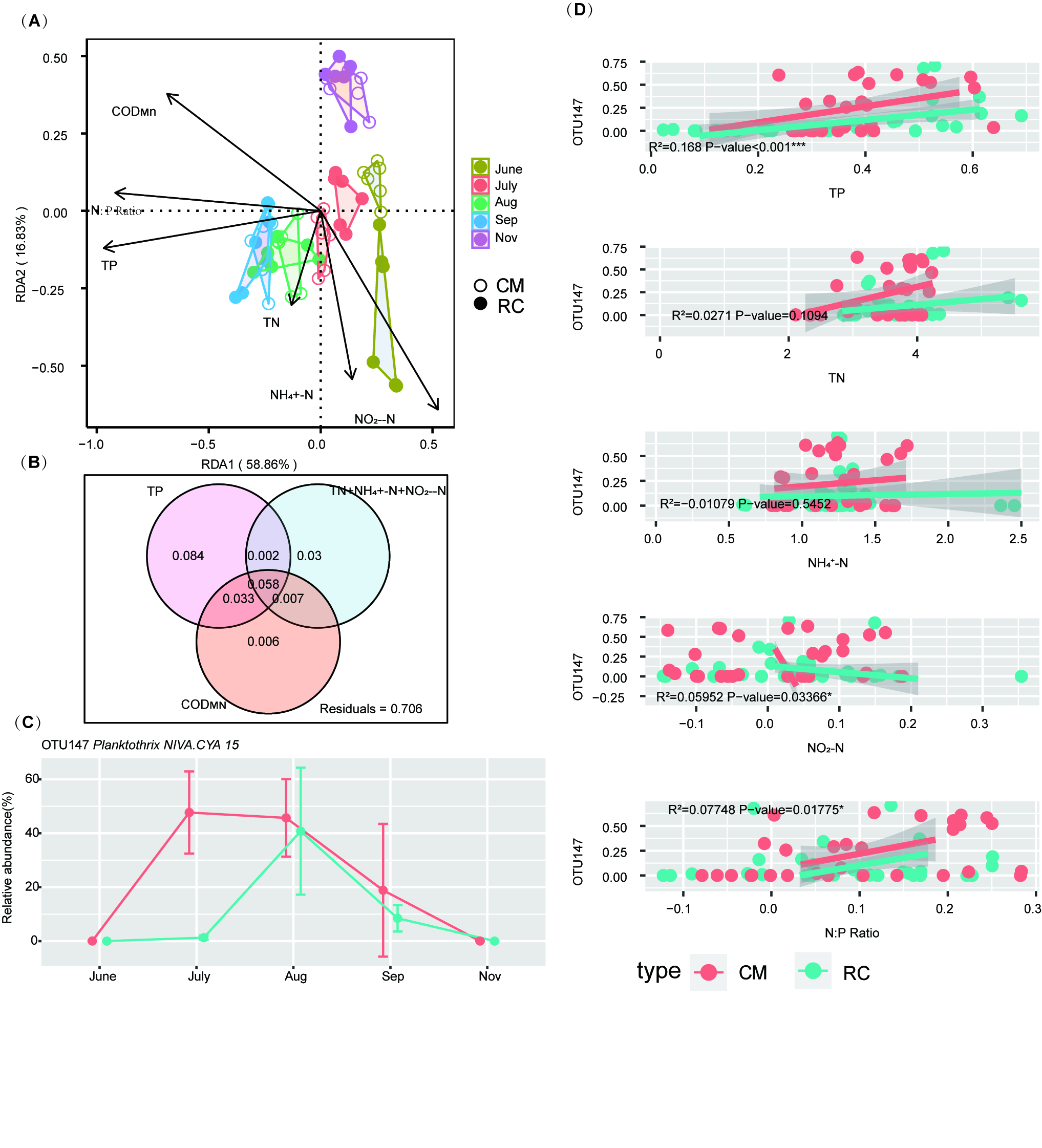

Supplement: Supplementary file 4 [file Image_1.TIF]

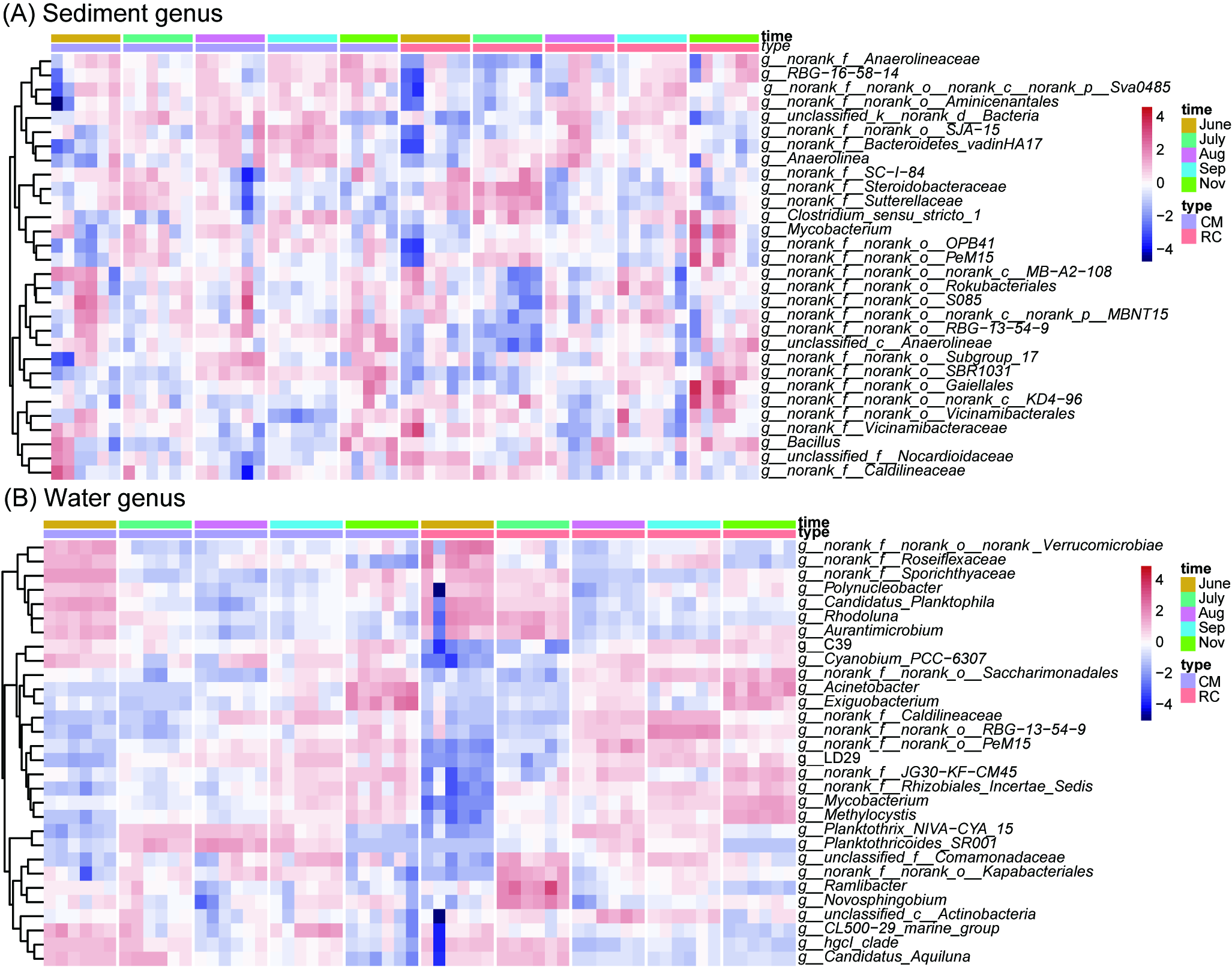

Supplement: Supplementary file 5 [file Image_2.TIF]

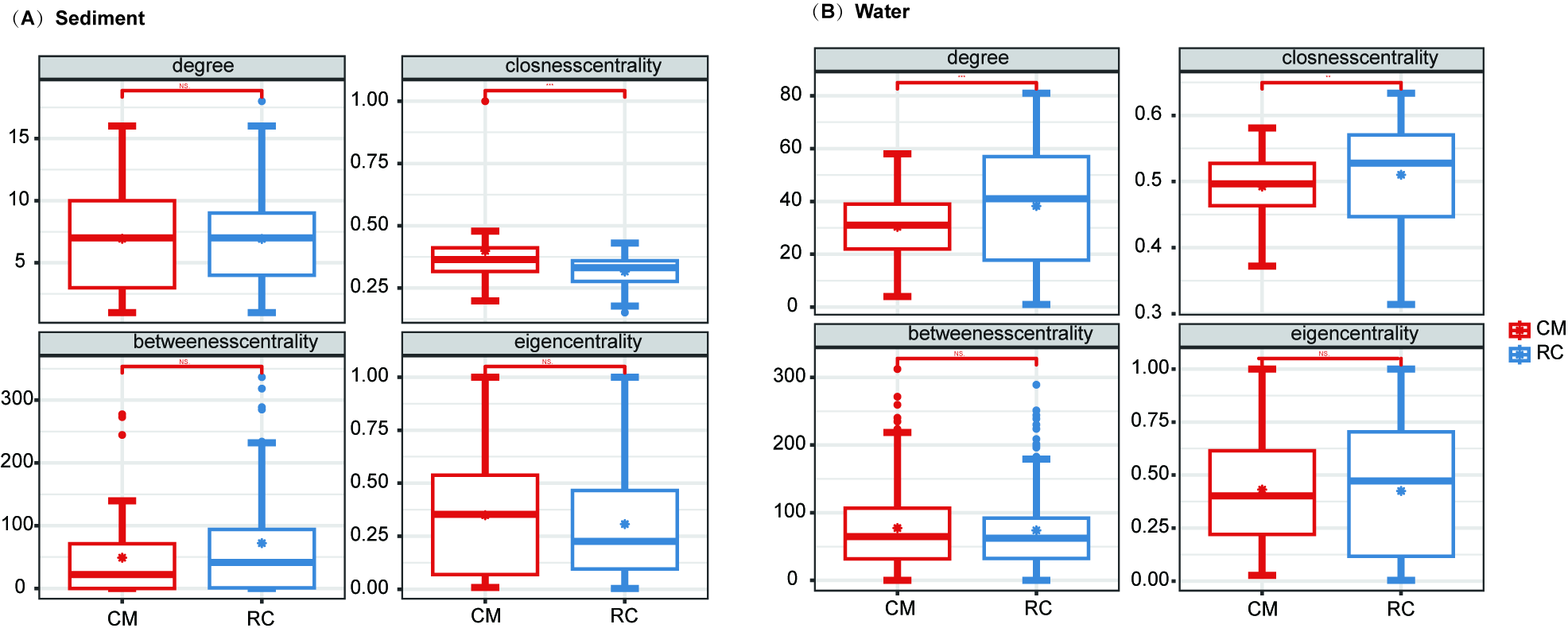

Supplement: Supplementary file 6 [file Image_3.TIF]
